# Real-Time Freezing of Gait Prediction and Detection in Parkinson’s Disease

**DOI:** 10.3390/s24248211

**Published:** 2024-12-23

**Authors:** Scott Pardoel, Ayham AlAkhras, Ensieh Jafari, Jonathan Kofman, Edward D. Lemaire, Julie Nantel

**Affiliations:** 1 Department of Systems Design Engineering, University of Waterloo, Waterloo, ON N2L 3G1, Canada; spardoel@uwaterloo.ca (S.P.); ayham.alakhras@uottawa.ca (A.A.); jkofman@uwaterloo.ca (J.K.); 2 School of Human Kinetics, Faculty of Health Sciences, University of Ottawa, Ottawa, ON K1N 6N5, Canada; ejafa099@uottawa.ca; 3 Faculty of Medicine, Ottawa Hospital Research Institute, University of Ottawa, Ottawa, ON K1H 8M2, Canada; elemaire@ohri.ca

**Keywords:** freezing of gait, plantar pressure, machine learning, wearable sensors, Parkinson’s disease, prediction

## Abstract

Freezing of gait (FOG) is a walking disturbance that can lead to postural instability, falling, and decreased mobility in people with Parkinson’s disease. This research used machine learning to predict and detect FOG episodes from plantar-pressure data and compared the performance of decision tree ensemble classifiers when trained on three different datasets. Dataset 1 (*n* = 11) was collected in a previous study. Dataset 2 (*n* = 10) included six new participants and four participants from Dataset 1 who were re-tested (approximately 2 years later), and Dataset 3 (*n* = 21) combined Datasets 1 and 2. The prediction model trained on Dataset 3 had a 2.28% higher sensitivity and 3.09% lower specificity compared to the models trained on Dataset 1. The model trained on Dataset 3 identified 86.84% of the total FOG episodes compared to 74.31% from the model trained on Dataset 1. Also, the model using Dataset 3 identified the FOG episodes 0.3 s earlier than the model developed with Dataset 1. The model trained using Dataset 3 showed improved performance in sensitivity, identification time, and FOG identification. The improvements using the expanded dataset (Dataset 3) in this study compared to the previous model reinforce the validity and generalizability of the original model. The model was able to predict and detect FOG well and is, therefore, ready to be implemented in a FOG prevention device.

## 1. Introduction

Parkinson’s disease (PD) is a neurodegenerative condition that affects the motor system due to a reduction in dopamine-producing neurons within the brain [1]. While PD symptoms may vary from person to person, one of the common and disabling motor symptoms in more advanced stages of the disease is freezing of gait (FOG) [2,3]. FOG is a walking disturbance that interferes with the ability to perform cyclical stepping activities and leads to an involuntary arrest of forward walking progression [1]. FOG has been described as feeling like one’s feet are ‘glued to the floor’ [4,5]. Freezing can also lead to overall reduced mobility and falling [4].

Rhythmic cues (i.e., cues administered at a constant rhythm in the form of auditory, visual, or tactile stimuli) can improve gait speed, reduce the number of freezing episodes, and increase the distance that people with PD can walk without experiencing FOG [6]. However, constant cues throughout the day, as well as cueing at a rhythm different from a person’s intended stepping rhythm, could be distracting and may even induce FOG [7]. Intelligent cues, which are administered only when needed, could reduce the disadvantages of constant rhythmic cueing. To enable intelligent cueing, it is necessary to first develop a system capable of detecting—or preferably—predicting FOG.

Detection and prediction of FOG episodes in real-time have been addressed through various methods, primarily leveraging wearable sensors [8,9,10,11,12,13,14]. Existing studies report varying sensitivities (63% to 100%) and specificities (59% to 100%) for FOG detection [8]. In [15], a convolutional neural network was used to predict FOG, on average, 2.72 s prior to onset, with a sensitivity of 84.61% and a specificity of 94.74%. This study classified FOG identification as a prediction. The study utilized acceleration data from the left leg and wrist, which may be difficult to acquire in a real-world device. In [16], a convolutional neural network employing a single inertial sensor placed on the waist achieved 87.7% sensitivity and 88.3% specificity. This model identified FOG episodes 1.2 s prior to onset. While many studies have focused on inertial measurement unit-based wearable devices [9,10,11,12,13,16,17,18], plantar-pressure sensors offer reduced measurement noise and added discretion in a real-world device. In [19], plantar-pressure data were utilized, yielding 96% sensitivity and 94% specificity for FOG detection. However, FOG episode predictive capabilities were not specified. While FOG identification methods provide valuable insights, their limitations lie in the reactive nature of detection, responding either during or after the onset of a freeze. Anticipating freezing events before they occur could enable timely intervention to prevent the onset of a freeze.

To further research FOG prediction using plantar-pressure data, this paper extends previous work [20,21] on the development of a FOG prediction and detection system using plantar-pressure data. The model in [20] was trained on data from only 11 participants with PD. A new dataset to train the model could have different FOG presentations, occurrence rates, and durations that can impact model performance. Furthermore, a larger number of participants would provide more varied data for the model training, which could improve performance overall. There is a need to determine if expanding the dataset would improve the model for greater generalizability and validate the model on a larger number of participants. In this paper, the number of participants was doubled, and analysis of the models trained on the different datasets was performed to help understand the generalizability of the models and to demonstrate greater model generalizability on a larger number of participants. Furthermore, outliers in the datasets were removed to analyze their effect on the generated models. This analysis will help in understanding the effect of different datasets on the proposed FOG prediction models. Such models could be implemented in intelligent cueing to enhance the mobility and safety of people with PD.

The rest of the paper is organized as follows: Section 2 outlines the design and implementation of the plantar-pressure sensor system, along with the data collection and analysis protocols. Section 3 presents the results, including the system’s accuracy, reliability, and performance metrics in real-time FOG detection. Section 4 discusses the implications and limitations of these findings, comparing the results from multiple datasets. Finally, Section 5 concludes with a summary of the contributions, potential applications, and recommendations for future research.

## 2. Materials and Methods

### 2.1. Participants

This study extends the model’s development and testing in [20]. The data collection methods are the same as in [20] and are briefly described here. For a previous study, data were collected from 11 participants (formally diagnosed with Parkinson’s disease by a neurologist), 7 of whom froze during testing (Table 1). In this paper, 4 participants from [20] were re-tested (nearly 2 years after they were first tested), and 6 new participants were tested (Table 2). Ethics approval was obtained from the University of Ottawa (H-05-19-3547) and the University of Waterloo (40954). A convenience sample of 6 new participants and 4 previously tested participants was recruited from the Ottawa-Outaouais community for this study. All participants provided informed consent.

The current paper explores the performance of a freeze prediction/detection model when trained using 3 groups of participants, referred to as Dataset 1, Dataset 2, and Dataset 3. Dataset 1 (*n* = 11) consisted of the initial 11 participants in [20]. Dataset 2 (*n* = 10) consisted of the second group of participants who were tested two years later: four of the participants from Dataset 1 and six new participants. Dataset 3 (*n* = 21) consisted of all participants from both groups (Datasets 1 and 2). Table 1 and Table 2 show the participant’s age and time since PD diagnosis at the time of testing.

Walking trial data were collected from 21 participants with PD. The participants for Dataset 2 were screened using the following criteria: experience freezing of gait at least once a day and able to walk the trial path unassisted. Participants performed walking trials at the Human Movement Performance Laboratory, University of Ottawa, while on their regular medication schedule. To increase the likelihood of FOG during walking, trials were scheduled prior to the participant’s next regularly scheduled medication dose, when possible. The New Freezing of Gait Questionnaire (NFOG-Q) [22] and the Unified Parkinson’s Disease Rating Scale (UPDRS) Motor Examination (Part III) [23] were used to assess the severity of FOG and PD motor symptoms.

The participants’ shoes were fitted with pressure-sensing insoles (FScan, Tekscan, Boston, MA, USA) for the walking trials. For each participant, a new set of FScan insoles was fitted to their shoes and calibrated according to the manufacturer’s instructions. Participants were asked to walk a freeze-inducing path [20] 30 times. When necessary to induce freezing, participants were asked to carry a tray with objects on top while performing a verbal task, which consisted of continuously saying words out loud beginning with a random letter.

### 2.2. Data Labeling

The plantar-pressure data and the videos were synchronized using a custom application written in MATLAB 2019b (The MathWorks Inc., Natick, MA, USA). The application used a one-foot stomp at the beginning of each trial as a synchronization event and several subsequent heel-strike events for verification. FOG episodes were labeled by researcher S.P. in consultation with researcher J.N. in cases of doubt. The labels were applied to the 30 Hz video data and were transferred to the 100 Hz plantar-pressure data using linear interpolation to the closest data point.

**Figure 1:**
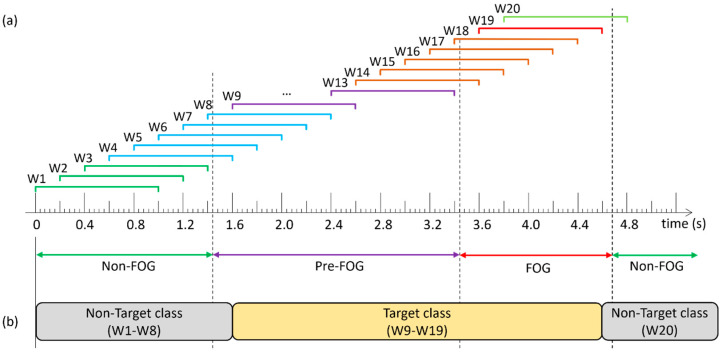
Example of data windowing and target class compositions: (**a**) Windows W1–W3 contain non-freezing of gait (FOG) data only; W4–W8: non-FOG and pre-FOG data; W9–W13: pre-FOG data only; W14–W18: pre-FOG and FOG data; W19: FOG data only; and W20: FOG and non-FOG data, (**b**) prediction model class composition [20].

As in [20], a FOG episode was defined as “the instant the stepping foot fails to leave the ground despite the clear intention to step” until “the instant the stepping foot begins or resumes an effective step”. An “effective step” was defined as a step where “the heel lifted from the ground, provided that it was followed by a smooth toe-off with the entire foot lifting from the ground and advancing into the next step without loss of balance”. As shown in Figure 1, each timestamp was labeled as either FOG or non-FOG, and a pre-FOG segment was defined as the 2 s immediately preceding the beginning of the FOG episode. The pre-FOG segment was set to be 2 s based on previous FOG prediction studies [18,20,24,25].

### 2.3. Windowing

The data were grouped into 1 s windows, which overlapped by 0.8 s (i.e., 0.2 s shift between consecutive windows, Figure 1). Machine learning models classified each window as the target class or non-target class. The target class was a FOG episode and the short period immediately prior. Purely FOG windows (W19) and pre-FOG windows (W9–W18) were assigned to the target class, and the non-FOG windows (windows that included any non-FOG data) (W1–W8, W20) were assigned to the non-target class.

### 2.4. Feature Extraction

Each data window was used to calculate features, which were passed to the FOG identification models for training. These features were based on the foot center-of-pressure (COP) position during the 1 s window, where X is the medial/lateral (ML) direction, and Y is the anterior/posterior (AP) direction. At any time, the COP coordinates were only considered valid if the total ground reaction force of one foot accounted for more than 5% of the total ground reaction force for two feet. This was done to remove erroneous COP position data caused by residual pressure data while the foot was in the swing phase. Following this check, the COP velocity was calculated as the first derivative of the COP position.

Based on previous research [20], the models used 4 features from each foot for a total of 8 features. One feature was in the time domain (number of AP COP reversals), and the other features were in the frequency domain. Fast Fourier transform was used to calculate the dominant frequency of the COP velocity in X (ML) and the dominant frequency of the COP velocity in Y (AP), and a wavelet transform was used to calculate the mean approximation coefficient power COP position Y (AP). All features were calculated for the right and left sides. The features are summarized in Table 3.

### 2.5. FOG Prediction Model Training

To facilitate dataset comparisons, all prediction models used the same parameters and training methods. Similar to those in [20], the models developed in the current paper were binary decision tree ensembles using random undersampling boosting (RUSBoosting). Each of the 100 trees had 5 splits. A leave-one-freezer-out cross-validation was performed, as in [25]. Participants who did not freeze were always included in the training dataset and never held out as test participants.

### 2.6. Performance Evaluation

**Figure 2:**
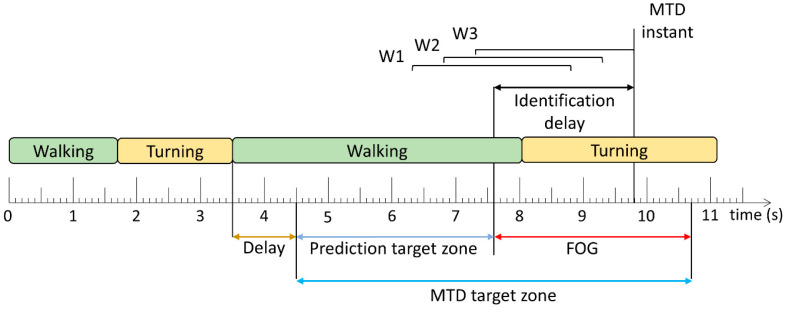
Model trigger decision (MTD) example. Three consecutive windows (W1–W3) classified as the target class (Figure 1) result in a MTD, where the MTD instant corresponds to the end of the third window. FOG is successfully identified (before or after onset) if a MTD instant occurs within the MTD target zone. The time difference between FOG onset and MTD instant is the identification delay (ID). The period between the beginning of the MTD target zone and the FOG onset is the prediction target zone. (Figure and caption adapted from [20]).

Two methods were used to evaluate the trained models: window-based and FOG episode-based. For the window-based evaluation, each window classification from the models was compared to the ground truth label to calculate sensitivity and specificity. The FOG episode-based evaluation determined if and when each episode was detected by the model. A model trigger decision (MTD) was defined as classifications of 3 consecutive windows as FOG (Figure 2). The time between FOG onset and successful MTD identification was the identification delay (ID). The ID was negative if the model predicted the episode before FOG onset and positive if the model detected the episode after onset.

The MTD target zone defines the time window during which the upcoming FOG can be predicted or detected. Before this zone, any MTD would likely not be related to the upcoming FOG, and after this zone, the FOG has ended and any MTD would be irrelevant. In [20] and this paper, the prediction target zone was set to a 6 s period initially. However, if another FOG episode, turn-to-walk transition, or stand-to-walk transition occurred within the initial prediction target zone, the prediction target zone’s length was adjusted to exclude FOG, turning, and standing data. The start of the prediction target zone was set to 1 s (shown as delay in Figure 2) after these turning and standing events so that false positives during turning or standing were not erroneously considered as early predictions of subsequent, unrelated FOG episodes.

Similar to [20], MTDs were classified into true positives (within the MTD target zone) and false positives (outside the MTD target zone). The false positive rate (false positives/walking trial) was calculated for each participant. Furthermore, for all datasets in this paper, false positive MTDs that occurred during standing or gait initiation were ignored. Gait initiation was defined as the first second of walking after standing. As a final stage in model development, a 2.5 s no-cue interval was used, wherein MTDs were ignored if they occurred less than 2.5 s after the previous MTD [25].

**Figure 3:**
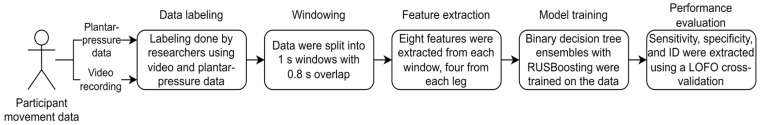
Flowchart of the data processing steps in this study.

The modeling approach shown in Figure 3, adapted from [20,21], was used to study the effects of expanding the dataset on model performance. To achieve this, three new models were trained: one on the existing dataset, one on a new dataset, and one on the combination of both datasets. This combination is possible because of the similarity of the data acquisition and walking path between datasets. The sensitivity, specificity, and ID from each model give a unique overview of the model performance on a micro level, exploring what kind of data most effectively impacts model performance.

## 3. Results

Table 4 and Table 5 present the NFOG-Q score, UPDRS-III score, and whether the participant froze during testing for Datasets 1 and 2. Table 6 and Table 7 show the number of trials, total trial duration, number of FOG episodes, total FOG episode duration, and total MTD target zone duration. Compared to Dataset 1, the average age of the participants for Dataset 2 was 1.7 years inferior, the average time since PD diagnosis was 0.45 years longer, the average NFOG-Q score was 5.1 points higher (maximum = 28), and the average UPDRS-III score was 0.9 points lower (maximum = 56). The standard deviation of age and time since PD diagnosis did not differ by more than 1.07 years. The NFOG-Q standard deviation differed by 0.1 points, and the UPDRS-III score standard deviation differed by 1.6 points. In Dataset 1, 7 of the 11 participants froze during testing, whereas in Dataset 2, all 10 participants froze during testing. Table 4 and Table 5 also show the change in NFOG-Q and UPDRS-III scores for participants in both Dataset 1 and Dataset 2 (P05, P07, P08, and P11). Given the degenerative nature of PD, it was expected that NFOG-Q and UPDRS-III would increase after 2 years, indicating a worsening of Parkinson’s symptoms. For participants P07 and P11, the NFOG-Q scores were indeed higher, indicating more severe freezing symptoms. For P11, this is also supported by not freezing during Dataset 1 testing but freezing during testing 2 years later. Based on the NFOG-Q score, participant P05 reported less freezing in the second questionnaire, but they did not freeze during the first data collection and did freeze during the second.

Table 8 shows the metrics used to evaluate model performance on the three datasets. The model trained on Dataset 2 had the highest sensitivity (82.50%), percentage of FOG episodes identified (94.86%), and the lowest ID (−1.10 s), though it had the lowest specificity (77.27%) of the three models. Dataset 1 had greater differences than Dataset 2 when both were compared with Dataset 3.

**Figure 4:**
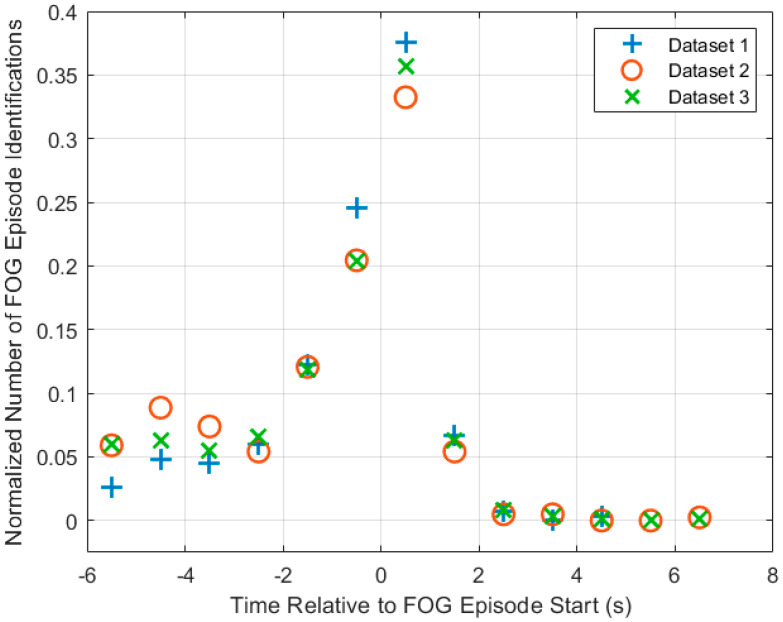
Normalized number of initial FOG identifications at each time interval plotted for each model. Normalization was performed by dividing the number of FOG episode identifications at each point for a dataset by the total number of FOG episode identifications for that dataset. The histogram was plotted with a fixed interval of 1 s starting at −6 s.

Figure 4 shows the distribution of FOG identifications relative to FOG onset. Dataset 1 shows the highest ID and the lowest relative number of identifications before onset. Dataset 2 has the lowest ID and shows the highest relative number of identifications before onset. Dataset 3’s performance lies mostly between Datasets 1 and 2.

**Figure 5:**
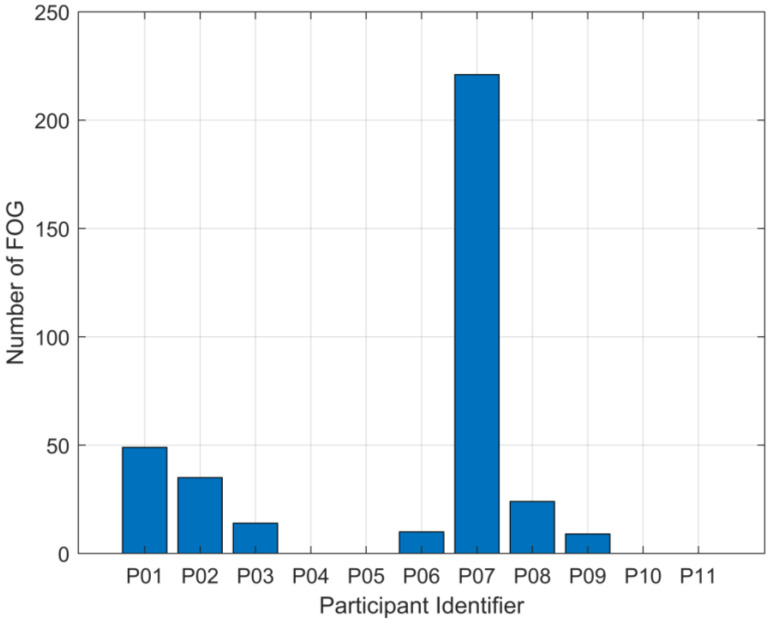
Total number of FOG occurrences for each participant. P07 shows a significantly higher number of FOG episodes compared to other participants in the dataset. P04, P05, P10, and P11 experienced no FOG episodes during testing.

To analyze possible outlier effects, outliers were removed from Datasets 1 and 3. The outliers include P07, who experienced FOG significantly more than the other participants, and non-freezers, who did not experience FOG during testing, as shown in Figure 5. The datasets were modified in three ways: P07 removed, all non-freezers removed, or both P07 and all non-freezers removed. Note that P07B (P07 was re-tested approximately 2 years later) did not have as many FOG occurrences or as much total FOG duration as P07 compared to their respective dataset and was thus not removed from the modifications. The results from these modifications are shown in Table 9.

**Figure 6:**
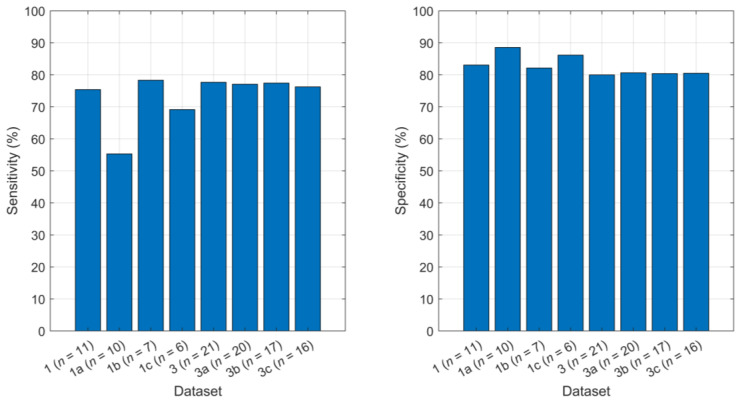
Change in sensitivity (**left**) and specificity (**right**) with each modification on Datasets 1 and 3.

As shown in Figure 6, removing P07 from Dataset 1 (Dataset 1a) caused sensitivity to drop by 20.09% from 75.40% to 55.31% and caused specificity to increase by 5.38% from 83.08% to 88.56%. Removing non-freezers from Dataset 1 (Dataset 1b) improved the performance compared to the model trained on Dataset 1. Sensitivity increased by 2.92% from 75.40% to 78.32%, and specificity dropped by 0.48% from 83.08% to 82.16%. When both P07 and non-freezers were removed from Dataset 1 (Dataset 1c), the model performance metrics were between the results for Datasets 1a and 1b, except for ID, which was higher and indicated later detection.

## 4. Discussion

The performance evaluation of the models across three datasets demonstrates their effectiveness in predicting FOG episodes. The models exhibit comparable performance to the existing methods in detecting and predicting FOG in a real-time system. This finding underscores the potential readiness of the model for integration into FOG identification and prevention systems, offering promising implications for enhancing care and quality of life for people who experience FOG.

The model trained on Dataset 2 outperformed the model trained on Dataset 1 in all metrics, and although there may be other contributing factors, there are two possible reasons for this difference. Firstly, the difference between Dataset 1 and the other datasets may have been caused in part by the presence of four participants who did not experience FOG during testing. Dataset 2, by comparison, had zero non-freezers. Secondly, P07, who is present in Dataset 1 but not Dataset 2, experienced 221 freezing episodes, while all other participants in the dataset combined experienced 141 freezes (14.1 freezes per participant excluding P07). P07’s total freeze time for all trials was 335.92 s, while all other participants combined experienced a total of 226.34 s (22.63 s of freezing per participant, excluding P07). Therefore, the increased severity and duration of P07’s freezing may have biased the model to prioritize identifying P07’s freezes over other participants.

Removing P07 from Dataset 1 (to produce Dataset 1a) adversely affected model performance. While the model became better at identifying true negatives and avoiding false positives, it was much worse at identifying true positives and avoiding false negatives. The model took less risk with its predictions, meaning it avoided false positives at the cost of missing true positives. This is most likely due to insufficient training data with FOG in the dataset. While RUSBoosting was used for all datasets to address the class imbalance usually present in FOG data, the method has limitations. Without sufficient target class data, undersampling and boosting cannot generate a well-performing model. In Dataset 3, which has more freeze data overall than Dataset 1, the effect of removing P07 was much less apparent. The model trained on Dataset 3a (P07 removed) outperformed the model trained on Dataset 3 in all metrics except for sensitivity, which dropped by only 0.61%. This shows that while P07’s data differ significantly from the other participants’ data, P07’s data contains many FOG instances and is, therefore, helpful for model performance when FOG instances in the training data are limited.

On the other hand, removing only non-freezers from Dataset 1 (to produce Dataset 1b) improved sensitivity by 2.92% with minimal effect on specificity. Interestingly, doing the same for Dataset 3 slightly worsened performance in all metrics, indicating that some non-freeze data were not detrimental to model performance when a dataset has a large amount of freeze data. In summary, the model trained on Dataset 1b (non-freezers removed) outperformed the model trained on Dataset 1, and the model trained on Dataset 3a (P07 removed) slightly outperformed the model trained on Dataset 3.

The composition of the training datasets significantly influenced model performance. Removing P07 from Dataset 1 led to a 20.09% drop in sensitivity but improved specificity by 5.38%, highlighting P07’s contribution to critical freeze data despite its distinctiveness. Conversely, removing non-freezers (Dataset 1b) improved sensitivity by 2.92% with minimal specificity impact, suggesting that non-freezers’ data diluted the model’s ability to focus on FOG patterns. These findings emphasize the need for balanced datasets that include diverse FOG severities and non-freezing behaviors to enhance both sensitivity and specificity. This demonstrates the importance of larger, more representative datasets for robust and generalizable models.

In all metrics and ID distribution (Figure 4), Dataset 3’s model performance was between the performance of Datasets 1 and 2 models because Dataset 3 is a combination of Datasets 1 and 2. However, the difference between the models was not large. Sensitivity and specificity varied by a maximum of 7.1% and 5.81% between models, respectively. In general, the models identified over 74% of FOG episodes and predicted over 40% of FOG episodes. The models identified FOG episodes before freezing onset by 0.64 s, 1.10 s, and 0.94 s for Datasets 1–3, respectively. The normalized ID distribution also had a nearly identical shape for all three models. The similarity of results across datasets indicated that the model trained on Dataset 3 was generalizable to predict FOG for people with PD who experience FOG once per day or more, thus supporting the readiness of the model for deployment in a FOG identification and prevention system.

Despite this study’s promising findings, there are some limitations. One such limitation is the size of the dataset. This study used data from 21 sessions and 17 participants to generate results. Even though the similarity between datasets signifies that the model is sufficiently trained, further increasing the number of participants could provide a more robust validation of the findings. Another limitation of the study is regarding the uncontrolled presence of various FOG presentations that may have influenced the results, as the most common manifestation in the data could have biased the analysis. Researchers have categorized FOG into three manifestations (also known as phenotypes) based on leg movement: akinesia, trembling, and shuffling steps [26]. Each of these categories of FOG has unique characteristics that may require different treatment. Exploring different classifications and developing a separate model for each type of FOG manifestation could yield more comprehensive insights, but this would require more participants. Further limitations stem from a finite amount of data for training the machine learning model. The model’s performance may have been affected by the imbalance between freezers and non-freezers in the training dataset. Optimizing the balance between these groups could mitigate potential biases in the model. Moreover, FOG is a symptom of mid to late-stage PD, meaning that the participants in this study have relatively higher disease severity compared to the broader PD population. Lastly, while the leave-one-freezer-out approach was employed for testing each model, evaluating a model’s performance on an entirely separate dataset would provide further validation of this study’s findings.

## 5. Conclusions

This paper presented an analysis of real-time FOG prediction and detection in PD using machine learning techniques. A viable model was developed and evaluated for FOG prediction and detection based on varying datasets created from 21 data collection sessions on 17 participants. This study highlights the value of expanding the dataset size and combining multiple datasets to improve the performance and robustness of FOG prediction models. Larger and more diverse datasets can significantly enhance the generalizability and accuracy of predictive models. These findings underscore the importance of exploring new datasets and methodologies in advancing the accuracy and effectiveness of FOG detection systems.

The results of this research demonstrate the feasibility and effectiveness of machine learning models to predict and detect FOG episodes based only on plantar-pressure data. The models showed good performance across multiple metrics, including sensitivity, specificity, FOG identification rates, and prediction lead times. The model trained on the largest dataset (Dataset 3) achieved a FOG identification rate of 86.84% and predicted FOG episodes on average 0.94 s before their onset. The model had 77.69% sensitivity and 79.99% specificity using leave-one-freezer-out cross-validation. These results suggest that the model has the potential to provide useful FOG instance information that can be used in a cueing device to help prevent FOG episodes and improve mobility for people with PD.

An in-depth analysis of the participants in the datasets revealed that the model performs best when trained on a dataset with a balance of freeze data between participants. Unbalanced datasets with too much or too little freezing hindered model performance. To address this, future models could be trained while limiting the amount of freezing data used from a single participant to reduce model bias. The number of non-freezers could also be limited to achieve the same effect. However, this should be done while maintaining substantial freezing and non-freezing data from freezers. Training the model with too little freezing data decreased model specificity and slightly increased the ID.

Furthermore, this study revealed that the proposed approach generated consistent and generalizable results for different datasets, indicating robustness and readiness for implementation in real-world applications. The comparison between datasets with and without specific participants highlighted the importance of data diversity in model training, as the presence of non-freezers or participants with varying degrees of FOG affected model performance.

Overall, this research contributes valuable insights into the development of a real-time FOG prediction and detection system, which could have a substantial effect on the quality of life for individuals living with PD. With further refinement and validation, the model could be integrated into wearable devices or smartphone applications to provide on-demand assistance and support for FOG management, ultimately enhancing mobility and safety for people with PD.

## Figures and Tables

**Table 1:** Dataset 1 participant information.

Participant Identifier	Age (Years)	Time Since PD Diagnosis (Years)
P01	67	16
P02	80	11
P03	71	11
P04	64	10
P05	70	14
P06	68	19
P07	78	5
P08	70	12
P09	80	10
P10	80	2
P11	72	5
Average (SD)	73.73 (5.80)	10.45 (5.01)

**Table 2:** Dataset 2 participant information.

Participant Identifier	Age (Years)	Time Since PD Diagnosis (Years)
P12	74	4
P13	66	19
P14	69	19
P15	81	3
P16	67	6
P17	58	11
P05 B ^1^	72	17
P07 B ^1^	81	8
P08 B ^1^	73	14
P11 B ^1^	74	8
Mean (SD)	71.50 (6.95)	10.90 (6.05)

^1^ ‘B’ indicates the second time this participant was tested.

**Table 3:** List of features used in FOG prediction models. Features from [20].

Feature Number	Feature Description
1	Number of AP COP reversals—Left
2	Number of AP COP reversals—Right
3	Dominant frequency of COP velocity in X (ML)—Left
4	Dominant frequency of COP velocity in X (ML)—Right
5	Dominant frequency of COP velocity in Y (AP)—Left
6	Dominant frequency of COP velocity in Y (AP)—Right
7	Mean approximation coefficient power of COP position Y (AP)—Left
8	Mean approximation coefficient power of COP position Y (AP)—Right

**Table 4:** Dataset 1 participant examination results.

Participant Identifier	NFOG-Q	UPDRS-III	Froze During Testing
P01	14	10	✓
P02	21	20	✓
P03	17	13	✓
P04	4	18	
P05	20	13	
P06	22	29	✓
P07	15	16	✓
P08	17	20	✓
P09	18	18	✓
P10	4	15	
P11	19	20	
Mean (SD)	15.5 (5.9)	17.5 (4.8)	

**Table 5:** Dataset 2 participant examination results.

Participant Identifier	NFOG-Q	UPDRS-III	Froze During Testing
P12	18	6.5	✓
P13	29	28	✓
P14	15	17	✓
P15	19	14.5	✓
P16	23	20.5	✓
P17	29	20	✓
P05B ^1^	15	-	✓
P07B ^1^	26	11	✓
P08B ^1^	10	10	✓
P11B ^1^	22	22	✓
Mean (SD)	20.6 (6.0)	16.6 (6.4)	

^1^ ‘B’ indicates the second time this participant was tested (approximately two years later).

**Table 6:** Dataset 1 participant testing results.

Participant Identifier	Number of Trials	Total Trial Duration (s)	Number of FOG	Total FOG Duration (s)	Total MTD Duration (s)
P01	26	881.4	49	33.81	98
P02	26	1199.9	35	92.4	99.4
P03	29	1456.09	14	14.84	91
P04	14	547.96	0	0	*
P05	24	1100.16	0	0	*
P06	29	1964.46	10	42.3	155.4
P07	26	2024.62	221	335.92	410.2
P08	22	1057.76	24	36.24	127.4
P09	28	1445.36	9	6.75	98
P10	29	1251.64	0	0	*
P11	29	1867.89	0	0	*
Mean (SD)	25.63 (4.29)	1345.20 (443.53)	32.91 (64.43)	51.11 (93.98)	154.20 (106.62)

* Since participants who did not freeze did not have a model evaluated using their data during the leave-one-freezer-out cross-validation, there were no MTD results for these participants.

**Table 7:** Dataset 2 participant testing results.

Participant Identifier	Number of Trials	Total Trial Duration (s)	Number of FOG	Total FOG Duration (s)	Total MTD Duration (s)
P12	12	457.20	9	9.90	57.40
P13	29	1436.37	41	67.24	207.20
P14	27	1472.58	23	63.25	233.80
P15	28	1353.80	34	93.84	*
P16	28	1872.08	28	370.72	*
P17	26	1907.36	83	678.94	516.60
P05B ^1^	23	1176.91	8	50.32	180.60
P07B ^1^	29	2268.96	126	496.44	676.20
P08B ^1^	29	1318.05	1	0.73	120.40
P11B ^1^	22	2461.80	75	324.00	*
Mean (SD)	25.3 (5.02)	1572.51 (549.48)	42.80 (39.95)	215.54 (224.77)	284.60 (208.79)

^1^ ‘B’ indicates the second time this participant was tested (approximately two years later). * Since participants who did not freeze did not have a model evaluated using their data during the leave-one-freezer-out cross-validation, there were no MTD results for these participants.

**Table 8:** Comparison of model performance in each dataset. FOG identified is the percentage of episodes identified before or after onset. FOG predicted is the percentage of episodes identified before onset. FOG detected is the percentage of episodes identified after onset. FOG predicted and detected is a percentage of the total FOG episodes, not of the FOG identified. FOG episodes may be both predicted and detected.

Dataset	Sensitivity (%)	Specificity (%)	FOG Identified (%)	FOG Predicted (%)	FOG Detected (%)	ID (s)
1 (*n* = 11)	75.40	83.08	74.31	40.61	51.93	−0.64
2 (*n* = 10)	82.50	77.27	94.86	57.01	80.14	−1.10
3 (*n* = 21)	77.68	79.99	86.84	49.11	68.48	−0.94
Mean (SD)	78.53 (2.96)	80.11 (2.37)	85.34 (8.46)	48.91 (6.70)	66.85 (11.57)	−0.89 (0.19)

**Table 9:** Model performance for modified datasets: (a) P07 removed, (b) non-freezers removed, and (c) P07 and non-freezers removed.

Dataset	Sensitivity (%)	Specificity (%)	FOG Identified (%)	FOG Predicted (%)	FOG Detected (%)	ID (s)
1 (*n* = 11)	75.40	83.08	74.31	40.61	51.93	−0.64
1a (*n* = 10)	55.31	88.56	59.57	30.50	40.43	−0.59
1b (*n* = 7)	78.32	82.16	79.28	44.48	53.31	−0.70
1c (*n* = 6)	69.17	86.17	78.01	37.59	52.48	−0.50
3 (*n* = 21)	77.68	79.99	86.84	49.11	68.48	−0.94
3a (*n* = 20)	77.07	80.66	89.98	53.25	72.58	−1.03
3b (*n* = 17)	77.42	80.39	85.95	47.97	67.85	−0.88
3c (*n* = 16)	76.27	80.52	89.81	53.08	71.53	−1.03

## Data Availability

The data will be made available from the authors upon reasonable request.
